# Phase II randomized trial of neoadjuvant metformin plus letrozole versus placebo plus letrozole for estrogen receptor positive postmenopausal breast cancer (METEOR)

**DOI:** 10.1186/1471-2407-14-170

**Published:** 2014-03-10

**Authors:** Jisun Kim, Woosung Lim, Eun-Kyu Kim, Min-Kyoon Kim, Nam-Sun Paik, Sang-Seol Jeong, Jung-han Yoon, Chan Heun Park, Sei Hyun Ahn, Lee Su Kim, Sehwan Han, Seok Jin Nam, Han-Sung Kang, Seung Il Kim, Young Bum Yoo, Joon Jeong, Tae Hyun Kim, Taewoo Kang, Sung-Won Kim, Yongsik Jung, Jeong Eon Lee, Ku Sang Kim, Jong-Han Yu, Byung Joo Chae, So-Youn Jung, Eunyoung Kang, Su Yun Choi, Hyeong-Gon Moon, Dong-Young Noh, Wonshik Han

**Affiliations:** 1 Department of Surgery, University of Ulsan College of Medicine, Asan Medical Center, Seoul, Korea; 2 Department of Surgery, Ewha Womans University School of Medicine, Seoul, Korea; 3 Department of Surgery, Korea Cancer Center Hospital, Korea Institute of Radiological & Medical Sciences, Seoul, Korea; 4 Department of Surgery, Seoul National University Hospital, 101 Daehakro, Jongno-gu, Seoul 110-744, Korea; 5 Department of Surgery, College of Medicine, The Catholic University of Korea, Seoul, Korea; 6 Chonnam National University Hwasun Hospital, Chonnam National University Medical School, Hwasun, Korea; 7 Department of Surgery, Breast and Thyroid Cancer Center, Kangbuk Samsung Hospital, Sungkyunkwan University School of Medicine, Seoul, Korea; 8 Division of Breast and Endocrine Surgery, Hallym University Sacred Heart Hospital, Hallym University College of Medicine, Anyang, Korea; 9 Department of Surgery, Inje University Sanggye Paik Hospital, Seoul, Korea; 10 Department of Surgery, Samsung Medical Center, Sungkyunkwan University School of Medicine, Seoul, Korea; 11 Center for Breast Cancer, National Cancer Center, Goyang, Republic of Korea; 12 Department of Surgery, Yonsei University College of Medicine, Seoul, Korea; 13 Department of Surgery, College of Medicine, Konkuk University, Seoul, Korea; 14 Breast Cancer Center, Department of Surgery, Gangnam Severance Hospital, Yonsei University College of Medicine, Seoul, Korea; 15 Department of Surgery, Inje University, Busan Paik Hospital, Busan, Korea; 16 Busan Cancer Center, Department of Surgery, College of Medicine, Pusan National University, Busan, Korea; 17 Department of Surgery, Seoul National University Bundang Hospital, Seoul National University College of Medicine, Seongnam, Korea; 18 Department of Surgery, Ajou University School of Medicine, Suwon, Korea; 19 Division of Breast and Endocrine Surgery, Kangdong Sacred Heart Hospital, Hallym University College of Medicine, Seoul, Korea; 20 Cancer Research Institute, Seoul National University College of Medicine, Seoul, Korea

**Keywords:** Metformin, Letrozole, Neoadjuvant, Estrogen receptor-positive Breast cancer

## Abstract

**Background:**

Neoadjuvant endocrine therapy with an aromatase inhibitor has shown efficacy comparable to that of neoadjuvant chemotherapy in patients with postmenopausal breast cancer. Preclinical and clinical studies have shown that the antidiabetic drug metformin has anti-tumor activity. This prospective, multicenter, phase II randomized, placebo controlled trial was designed to evaluate the direct anti-tumor effect of metformin in non-diabetic postmenopausal women with estrogen-receptor (ER) positive breast cancer.

**Methods/Design:**

Patients meeting the inclusion criteria and providing written informed consent will be randomized to 24 weeks of neoadjuvant treatment with letrozole (2.5 mg/day) and either metformin (2000 mg/day) or placebo. Target accrual number is 104 patients per arm. The primary endpoint will be clinical response rate, as measured by calipers. Secondary endpoints include pathologic complete response rate, breast conserving rate, change in Ki67 expression, breast density change, and toxicity profile. Molecular assays will be performed using samples obtained before treatment, at week 4, and postoperatively.

**Discussion:**

This study will provide direct evidence of the anti-tumor effect of metformin in non-diabetic, postmenopausal patients with ER-positive breast cancer.

**Trial registration:**

ClinicalTrials.gov Identifier NCT01589367

## Background

Metformin, which is commonly used to treat type 2 diabetes, is a relatively safe drug with known pharmacokinetics and manageable toxicities. In addition, numerous experimental, epidemiologic, observational, and clinical studies have shown that metformin has anti-tumor effects [1]. For example, in a preclinical mouse xenograft model, metformin reduced the effective dosages of standard chemotherapeutic drugs and had preferential effects on tumorigenic cells [2]. A retrospective clinical study showed that patients taking metformin during neoadjuvant chemotherapy had a higher pathologic complete response (pCR) rate than diabetic patients not taking metformin or non-diabetes patients (24% vs 8% vs 16%, p = 0.02) [3,4]. Two potential mechanisms of anti-cancer action of metformin have been suggested. First, metformin may directly activate adenosine monophosphate kinase (AMPK), resulting in the downstream inhibition of mTOR signaling and the consequent suppression of cell proliferation [5-7]. Second, metformin-induced decreases in circulating insulin and insulin-like growth factor (IGF) concentrations may reduce the activation of the IGF-receptor signaling axis, resulting in decreases in growth promotion and mitogenesis [8-10]. Thus, the anti-cancer effects of metformin are mediated through a systemic improvement in metabolic profile and by directly targeting tumor cells [11,12].

Questions remain, however, about the clinical benefits of metformin as an anti-cancer agent in patients with breast cancer. Although one large-scale, phase III trial of adjuvant metformin has been initiated in women with breast cancer (NCIC CTG MA.32) [1], the accrual and treatment process is still ongoing, and several years of follow-up are needed to determine survival benefits. In addition, little is known about the effects of metformin on different subtypes of breast cancer or on the synergy between metformin and concurrently administered systemic agents. In addition, the optimal dosage of metformin that shows maximal anti-tumor effects with acceptable toxicities has not been determined. In this context, neoadjuvant treatment is the most efficient setting to assess the short-term in vivo effects of drug therapy in breast cancer patients. Neoadjuvant endocrine therapy results in a comparable response but lower toxicity compared with neoadjuvant chemotherapy in women with ER-positive breast cancer [13]. In postmenopausal women, aromatase inhibitors are associated with higher response rates than tamoxifen [14,15]. A recent phase II trial found that neoadjuvant treatment with everolimus, an mTOR inhibitor, plus letrozole resulted in a better response rate than letrozole alone [16]. In addition, neoadjuvant metformin was shown to lower Ki67 level [17]. These results suggested that metformin may be effective, when combined with letrozole, in postmenopausal women with ER-positive breast cancer. We therefore designed and initiated a phase II clinical trial evaluating the anti-tumor effect of neoadjuvant metformin in postmenopausal women with ER-positive breast cancer by comparing treatment with letrozole plus metformin or placebo.

## Methods/Design

### Study goal

The goal of this study was to evaluate the benefits of combining metformin and letrozole in the neoadjuvant treatment of postmenopausal women with ER-positive breast cancer. The primary end point is the rate of tumor response (clinical response rate) at 24 weeks, determined by measurements with calipers; changes in size on ultrasound, mammography, and MRI will be used as secondary efficacy assessments. Clinical response includes complete response (CR) and partial response (PR) by RECIST v1.1 criteria.

### Study design

METEOR is a phase II, prospective, randomized, double-blinded, placebo-controlled multicenter clinical trial. Twenty-one centers belonging to the Korean Breast Cancer Society Study Group are participating in this study (KBCSG-013). Patients will be randomly assigned (1:1) to receive letrozole 2.5 mg/day plus either metformin or placebo for 24 weeks before surgery. The initial dose of metformin will be 1000 mg/day for the first week, followed by 1500 mg/day for the second week, and 2000 mg/day from the third week onward. Patients will be randomized sequentially, stratified by center, with randomization codes. Block randomization will be used (SAS 9.2) in cooperation with the Medical Research Collaborating Center (MRCC) of Seoul National University Hospital. All study personnel will be masked to treatment. This study protocol has been approved by the Korea Food and Drug Administration (KFDA) as well as the institutional review board of each center, and was registered at clinicaltrials.gov (NCT 01589367). Written informed consent was obtained from all participants.

### Eligibility criteria and sample size calculation

Eligible patients are postmenopausal women with histologically confirmed ER-positive, stage II or III, primary breast cancer with palpable and clinically measureable tumors. The target population is 208 women, 104 in each arm. Sample size was calculated based on expected clinical response rates (cRR) of 55% in the letrozole plus placebo arm and 70% in the letrozole plus metformin arm, with α = 0.10 and 80% power and including an estimated 10% drop out rate. ER positivity is defined as ≥10% nuclear staining by immunohistochemistry or Allred score ≥3. Criteria for determining menopause include bilateral oophorectomy, age ≥60 years, or age <60 years and amenorrhea for ≥12 months and FSH >30 mIU/ml. Patients with diabetes (HbA1c ≥6.5% or fasting plasma glucose (FPG) ≥126 mg/dL (≥7 mM)); clinical T4, N3, or M1 disease; bilateral cancer, or inflammatory breast cancer will be excluded, as will patients with a history of lactic acidosis or at high risk of having metformin-induced lactic acidosis, such as those with high alcohol consumption or NYHA class III/lV congestive heart failure.

### Treatment schedule and evaluation

**Figure 1:**
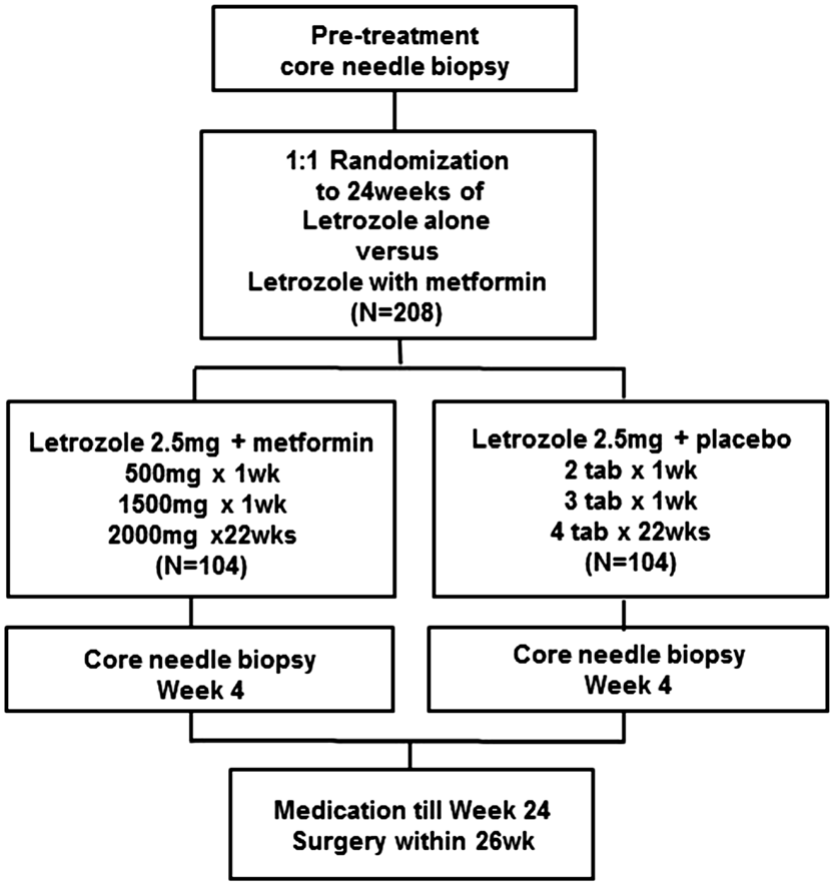
**Scheme of the METEOR trial.** Patients meeting the inclusion criteria will be 1:1 randomized to 24 weeks of letrozole plus metformin or letrozole plus placebo. Core needle biopsies will be recommended after 4 weeks of treatment. Surgery will be performed within 2 weeks after the end of the 24 week treatment period.

Patients will be randomly assigned (1:1) to 24 weeks of neoadjuvant treatment with metformin plus letrozole or placebo plus letrozole (Figure 1). Medications will be distributed at each monthly visit, with compliance assessed by counting the remaining tablets.

Tumor size will be measured clinically with calipers before treatment and sequentially every month. Each patient will undergo a careful physical examination at each monthly visit, and patients with progressive disease (PD) will be discontinued from the study and scheduled for immediate surgery. Patients with stable disease (SD) will be continued on treatment. Ultrasound-guided core needle biopsy samples will be obtained at the first visit and after four weeks of medication, with Ki67 levels in these samples centrally assessed. It is also recommended that 4-week blood samples be obtained to measure serum biomarker concentrations. Surgery will be scheduled within 2 weeks after completing the 24 weeks of medication. Tissue from surgical specimens will be collected for further planned assessments of biomarkers by paired analysis with the pretreatment and week 4 core needle biopsy specimens. HbA1c, FPG, insulin, c-peptide, and IGF-1 concentrations will be measured at baseline and after 12 and 24 weeks of treatment. Mammography, ultrasonography, and bilateral breast MRI will be performed before starting medication and after completion of treatment just before surgery.

### Analysis of the results

The primary endpoint is cRR. Tumor response will be assessed by RECIST criteria v1.1. Tumor size will be measured by individual clinicians at monthly visits. The secondary endpoint is pathologic complete response (pCR), defined as the absence of invasive cancer at the primary site and in the axilla. Before treatment, each patients will be categorized by surgeons into one of three groups: 1) marginal for breast conservation, 2) candidate for mastectomy only, and 3) inoperable by standard mastectomy [18]. Baseline assessment and actually performed surgery will be compared. The breast conservation rate in each arm will be evaluated, and the toxicity profile of each arm will be assessed every 4 weeks using NCI-CTCAE version 4.0 (http://ctep.cancer.gov/reporting/ctc.html). All parameters will be collected and managed using an e-clinical trial platform (MEBICA™).

### Translational research project

In addition to assaying Ki-67, phosphorylated S6 kinase 1 (p-S6K1) will be analyzed by immunohistochemistry (IHC) in a central laboratory. Fresh frozen tissue of week 4 biopsies and final surgical specimens will be collected from several major hospitals participating in this study. These samples will be used for DNA microarray analysis or whole transcriptome sequencing using next generation sequencing (NGS) technology. Phosphorylation of AMPK(T172), expression of IR(insulin receptor) and OCT1(Organic cation transporter) will be measured. Assessment of apoptosis will be done by Miller-Payne Grading system along with various commercialized kits.

We previously reported that breast density reduction after short term adjuvant endocrine therapy was predictive of recurrence-free survival [19]. We intend to analyze the association between breast density reduction and response to endocrine therapy in this prospective trial. Pre- and post-medication mammography results and breast MRI images of all patients will be centrally reviewed. Breast density will be measured centrally using computer-assisted software, Cumulus (University of Toronto, Toronto Ontario, Canada), by a single observer. The density of both breasts will be assessed by breast MRI [20], with the cranio-caudal view of the contralateral breast used as a reference to evaluate the percent change in mammographic density.

## Discussion

The maximum effective dose of metformin to treat hyperglycemia in patients with type 2 diabetes is 1000 mg twice daily. However, the metformin dose that yields the maximal anti-tumor effect is unclear. Results from two xenograft models reported that the human equivalent of 1500–2250 mg/day was needed to inhibit tumorigenesis [21-23]. A preoperative window of opportunity trial in breast cancer patients utilized a dose of 2000 mg/day [24], whereas the ongoing NCIC CTG MA32 phase III clinical trial is testing the effect of adjuvant metformin, is utilizing a dosage of 1700 mg/day. To use the maximum dose of metformin in this neoadjuvant setting, as well as to ensure patient safety, the dose is gradually increased over the first few weeks of treatment, and doses may be adjusted in response to toxicities/adverse events. The results of this trial will provide important information on the optimally effective and safest dose of metformin in non-diabetic breast cancer patients.

This study was designed to evaluate the direct anti-tumor effects of metformin in human breast cancer cancers. We expect that metformin will show a synergy with neoadjuvant letrozole in ER-positive breast cancer patients similar to that of the mTOR inhibitor, everolimus. Parallel translational research may provide a better understanding of the mechanism of action of metformin in cancer and may reveal biomarkers predictive of response to metformin.

## Abbreviations

ER: Estrogen receptor; AMPK: Adenosine monophosphate kinase; pCR: Pathologic complete response; mTOR: mammalian target of rapamycin; IGF: Insulin-like growth factor; CR: Complete response; PR: Partial response; RECIST: Response evaluation criteria in solid tumors; cRR: clinical response rate; PD: Progressive disease; SD: Stable disease; MRI: Magnetic resonance image; p-S6K1: phosphorylated S6 kinase 1; NGS: Next generation sequencing.

## Competing interests

All authors declare that they have no competing interests.

## Authors’ contributions

JK and WH drafted the manuscript and wrote the original protocol for the study. All authors participated in the design of the study. JK filed for ethical approvals from the KFDA and registered the trial on clinicaltrials.gov. WL performed the statistical analysis. EK designed the molecular study. MK was involved in the pharmaceutical process of SNUH and the process of patient enrollment. DN directed the entire process. All authors read and approved the final manuscript and are proceeding with the study in their respective centers.

## Pre-publication history

The pre-publication history for this paper can be accessed here:

http://www.biomedcentral.com/1471-2407/14/170/prepub
